# Simplified All-In-One CRISPR-Cas9 Construction for Efficient Genome Editing in *Cryptococcus* Species

**DOI:** 10.3390/jof7070505

**Published:** 2021-06-24

**Authors:** Ping Zhang, Yu Wang, Chenxi Li, Xiaoyu Ma, Lan Ma, Xudong Zhu

**Affiliations:** 1 Beijing Key Laboratory of Genetic Engineering Drug and Biotechnology, College of Life Sciences, Beijing Normal University, Beijing 100875, China; zp1516@bnu.edu.cn (P.Z.); lcx1219@163.com (C.L.); mxyaxx@163.com (X.M.); m1838572627@163.com (L.M.); 2 College of Life Sciences, Jiangxi Agricultural University, Nanchang 330045, China; wangyu08063115@163.com

**Keywords:** *Cryptococcus neoformans*, *Cryptococcus deneoformans*, CRISPR-Cas9 system, BspQI

## Abstract

*Cryptococcus neoformans* and *Cryptococcus deneoformans* are opportunistic fungal pathogens found worldwide that are utilized to reveal mechanisms of fungal pathogenesis. However, their low homologous recombination frequency has greatly encumbered genetic studies. In preliminary work, we described a ‘suicide’ CRISPR-Cas9 system for use in the efficient gene editing of *C. deneoformans*, but this has not yet been used in the *C. neoformans* strain. The procedures involved in constructing vectors are time-consuming, whether they involve restriction enzyme-based cloning of donor DNA or the introduction of a target sequence into the gRNA expression cassette via overlap PCR, as are sophisticated, thus impeding their widespread application. Here, we report the optimized and simplified construction method for all-in-one CRISPR-Cas9 vectors that can be used in *C. neoformans* and *C. deneoformans* strains respectively, named pNK003 (Genbank: MW938321) and pRH003 (Genbank: KX977486). Taking several gene manipulations as examples, we also demonstrate the accuracy and efficiency of the new simplified all-in-one CRISPR-Cas9 genome editing tools in both Serotype A and Serotype D strains, as well as their ability to eliminate Cas9 and gDNA cassettes after gene editing. We anticipate that the availability of new vectors that can simplify and streamline the technical steps for all-in-one CRISPR-Cas9 construction could accelerate genetic studies of the *Cryptococcus* species.

## 1. Introduction

The basidiomycete yeasts *Cryptococcus neoformans* and *Cryptococcus deneoformans* are opportunistic human pathogens that mainly infect immunocompromised individuals, particularly those with AIDS or who have received organ transplant surgery [1]. The *C. neoformans* species complex has also been well studied as a model of fungal pathogenesis [2,3]. Two different transformation systems, biolistic and electroporation transformation, have been developed to disrupt a number of genes via homologous recombination (HR) [4,5]. Yet, the low homologous recombination frequency obtained with these methods still remains a major hurdle to accelerate the pathogenic study of this fungus. The HR frequency in the Serotype D strain falls between 1% and 4%, even with the most expensive biolistic method [6]. Although this number is much higher than that obtained by electroporation (1/10-3 to 1/10-5), the extremely low HR frequency dramatically hinders the efficient analysis of gene function, especially for researchers with no access to a biolistic machine [7,8,9,10].

As an adaptive immune mechanism in prokaryotes, the CRISPR (clustered regularly interspaced short palindromic repeats) and Cas9 (CRISPR-associated RNA-guided DNA endonuclease) system are the latest gene editing technologies to be developed. They take advantage of the simple design of a single crRNA:tracrRNA chimeric guide RNA (gRNA) [11]. gRNA includes 17–20 base pairs of target sequences that produce double-strand breaks by Cas9 at the target locus, which triggers gene editing by NHEJ (non-homologous end joining) or homologous recombination repair [12]. The CRISPR-Cas9 system has been acclaimed for its high level of efficiency in editing the genomes of a series of organisms, particularly in organisms that are refractory to genomic manipulation with traditional methods [13,14,15,16], including *Cryptococcus* species. Three different CRISPR-Cas9 systems involving electroporation into yeast cells and one involving a biolistic transformation method have been used in *C. deneoformans* and *C. neoformans* strains (reviewed by Morio et al., 2020) [17]. In addition to the ‘suicide’ CRISPR-Cas9 vector established by our lab, which contains all functional elements in one plasmid [18], Fan et al. reported a Transient CRISPR-Cas9 coupled with an electroporation (TRACE) system that separates Cas9 and gRNA and co-transforms the gene deletion cassettes into yeast cells by electroporation [19]. Further, Wang et al. described the delivery of preassembled CRISPR-Cas9-gRNA ribonucleoproteins (RNPs) via electroporation to generate genome editing in *Cryptococcus* species [20].

Despite the high transformation rate of the unique ‘suicide’ CRISPR-Cas9 vector by electroporation, the cloning procedures used for introducing the target sequence and donor DNA into the pBS-URA5-CRISPR vector are time-consuming and complex [18], which may limit its general applications. We, therefore, developed a new vector for the *C. deneoformans* strain named pRH003 and another vector for the *C. neoformans* strain named pNK003 in order to greatly simplify the procedure of CRISPR-Cas9 construction. Two reverse BspQI restriction sites enabling simple directional cloning of a pair of annealed oligonucleotides scarlessly into the vector before the gRNA scaffold were introduced. The oligonucleotides needed to be designed as 5′-TTGN19(targe-site sense strand)-3′ and 5′-AACN19(target-site antisense strand)-3′, respectively. The guanine nucleotide of the 5′ terminus of the target sequence was used as the transcription initiation signal. Our previous results indicate that constitutive expression of both Cas9 and gRNA is toxic to *C. deneoformans*. A similar phenomenon has also been observed in *Saccharomyces cerevisiae* [21] and *Schizosaccharomyces pombe* [22] when using this system. Interestingly, the results from Samantha et al. indicated that the CRISPR-Cas9 system does not affect the growth and virulence of *C. neoformans* Serotype A strain H99 [23]. One possible reason for this is that promoters with different expression intensities were used to drive the expression of CRISPR-Cas9. However, considering that constitutive expression of Cas9 and gRNA could increase the chances of off-target effects and preventing subsequent reintroduction of the wild type genes, it is believed to be a better option for eliminating Cas9 and the gDNA cassette after gene editing, regardless of whether they are toxic to host cells [24].

Therefore, in addition to the hybridized oligonucleotides that are cloned directionally into the gRNA expression cassette through two reverse BspQI restriction sites, we also improved the procedures of introducing two homology arms with in-fusion cloning technology in a single step [25] to eliminate Cas9 and gDNA cassettes spontaneously after gene editing according to the principle of positive and negative selection [26]. As a restriction enzyme- and ligation-independent strategy for cloning, In-fusion cloning technology enables directional, seamless cloning of multiple inserts into any linearized vector in a single 15-min reaction. Thus, new procedures greatly shorten the amount of time needed to construct an HR deletion structure and break the constraints of restriction sites. Taking several genes as examples, our results revealed that the simplified all-in-one CRISPR-Cas9 vectors pRH003 and pNK003 are highly efficient for introducing DSBs (double-strand break) at target loci in both Serotype A and Serotype D strains. Subsequently, they stimulated the repair of DSBs with NHEJ or HR. We expect that these new plasmids will further facilitate the widespread use of the CRISPR-Cas9 system in *Cryptococcus* species.

## 2. Materials and Methods

### 2.1. Strains, Media and Reagents

*Cryptococcus neoformans* strain H99 (Serotype A) and *Cryptococcus deneoformans* strain JEC21 (Serotype D) were used as the wild types. The uracil auxotrophic strain of JEC21 used was 4500FOA. Yeast cells were grown in YPD medium (a rich medium that contains 1% yeast extract, 2% peptone, and 2% glucose at pH 6.0) or YNB medium (a basal selection medium that contains 0.17% yeast nitrogen base without ammonium and amino acids, 0.5% ammonium sulphate, and 2% glucose at pH 6.2). Solid media contained 2% agar. All yeast cultures were incubated at 30 °C unless indicated. All plasmids used for this study were maintained in *Escherichia coli* DH5α cells grown at 37 °C in Luria–Bertani broth or agar supplemented with 100 mg L^−1^ ampicillin.

T4 DNA ligase, the in-fusion cloning kit, and most of the restriction enzymes used for cloning were obtained from TAKARA/Clontech (Dalian, China). All quick-cut enzymes used for the restriction enzyme digestion analysis were obtained from MBI Fermentas (Shenzhen, China). The BspQI restriction enzyme used for linearizing the pRH003 and pNK003 vectors was obtained from NEB (Beijing, China). The FastPfu high-fidelity polymerase used for PCR cloning was obtained from TransGen Biotech (Beijing, China) and the 2×Es Taq MasterMix used for PCR verification was obtained from Cwbiotech (Beijing, China). A DNA clean kit and DNA gel purification kit were obtained from Corning Axygen (Jiangsu, China). All sequencing and primer synthesis processes were conducted by or purchased from GeneWise (Tianjin, China). All primers used in this study are listed in Table S1.

### 2.2. Construction of pRH003 and pNK003 Plasmids

In order to obtain a suitable cloning vector, the primer pair pX-F/pX-R was used to amplify the backbone region of pX330 (Addgene plasmid 42230), which contains the pBR322 origin of replication and the ampicillin resistance gene. PCR with the primer pair pT3-F/pT7-R was used to obtain the MCS-URA5 region (MCS, multiple cloning site; *URA5*, a marker for transformation) from the pBS-URA5 plasmid. The two purified fragments were assembled with the in-fusion cloning method to produce the pRH001 plasmid. For the construction of the gRNA expression cassette to produce single gRNA, overlap PCR was used to ligate the CnU6 promoter, double BspQI restriction sites, guide RNA scaffold, and a “TTTTTT” transcription terminator. The resulting gDNA cassette product was digested with XhoI/ClaI and ligated into pRH001 to obtain the pRH002 plasmid. Finally, an intact Pact:Cas9 expression cassette in the pBS-URA5-CRISPR vector [18] was cut with XbaI/NotI and inserted into pRH002 to obtain the pRH003 plasmid (Figure S1). Furthermore, to facilitate the virulence studies, pHYG003 plasmid which contains the selection marker hph to confer resistance to Hygromycin B in the Serotype D strain was constructed on the basis of pRH003.

In a similar manner, the pNK003 plasmid used for *C. neoformans* strain H99 was generated on the basis of pRH003. Firstly, the pRH003 plasmid was digested with XbaI/NcoI to remove the JEC21 *ATC1* promoter. The *TEF1* (CNAG_06125) promoter amplified with the primer pair pTEF1-F/pTEF1-R was utilized to replace the JEC21 *ACT1* promoter on pRH003 and start the expression of the Cas9 cassette in the H99 strain. The plasmid was named pNK001. Secondly, the H99 U6 promoter was inserted to initiate gRNA expression. The primer pairs H99CnU6-F/BspQI-H99CnU6-R and BspQI-gRNA-F/gRNA-R were implemented to obtain the H99 U6 promoter and gDNA fusion cassette in which double BspQI restriction sites were included to clone the target sequence, and the new gDNA cassette was ligated into the pNK001 plasmid via XbaI/NcoI restriction endonucleases. Thus, we obtained the pNK002 plasmid. Finally, the hph marker, which contained the H99 *ACT1* promoter and the *IGPS* (CNAG_04501) terminator, was inserted into the pNK002 plasmid through digestion with HindIII/BamHI restriction endonucleases and conferred resistance to Hygromycin-B in the H99 strain. Thus, we obtained the pNK003 CRISPR-Cas9 plasmid (Figure S1). The correctness of all the constructed plasmids was confirmed by restriction digestion and sequencing. The nucleotide sequence data of pNK003 and pRH003 were deposited into GenBank with the accession numbers MW938321 and KX977486, respectively.

### 2.3. Cloning of the Target Sequence into the gRNA Cassette

pRH003 and pNK003 CRISPR-Cas9 vectors both contain two reverse BspQI restriction sites between the U6 promoter and the gRNA component. The hybridized target DNA with 5′ overhangs can be conveniently introduced into the BspQI-cut plasmid. Plasmid can be digested with BspQI (NEB, Beverly, MA, USA) at 50 °C for 20–30 min in accordance with the operating manual. When digestion is complete, column-purified linearized plasmid can be stored at −20 °C for long periods. The stock of digested vector can be used for cloning any target sequences.

Taking the H99 ADE2.Z target site for example, a pair of oligos, H99ADE2.Z-F H99ADE2.Z-R, were annealed in a PCR Thermocycler at 95 °C for 5 min. Then, the PCR tube was taken out and left on the bench at room temperature to cool down for more than one hour. After annealing, the hybridized oligo was diluted to 1:50 with double-distilled water. Hybridized oligonucleotide substrates containing the target sequences and 5′ overhangs were then directly ligated into the BspQI-digested pNK003 vector. The ligation reaction was incubated at 4 °C for 4 h. Then, it was transformed into *E. coli* DH5α competent cells. After incubating the ampicillin-containing agar plates at 37 °C for 14–16 h, six random colonies were picked to verify gDNA cassette sequences with the T3 universal primer to confirm the successful insertion of the ADE2.E target sequence into the gRNA expression cassette. Three targets for the Serotype D strain and two for Serotype A used in this study were designed and optimized with sgRNAcas9 software [27]. This software can scan putative off-target sites and minimize the possibility of unintended off-target effects across the whole genome scale.

### 2.4. Introduction of Donor DNA with the In-Fusion Cloning Technique

In the optimized method, the in-fusion cloning technique was applied to insert two homology arms surrounding the selection marker simultaneously without the need to consider the restriction sites. For both the pRH003 and pNK003 plasmids, three common unique restriction sites flanking the marker that could be used to linearize the vector were designed: ClaI, BamHI, and SpeI. Taking the example above, after cloning the ADE2.E target sequence, the pNK003/ADE2.E plasmid was digested with ClaI/BamHI, the two restriction sites flanking the hph selectable marker. Then, the hph selectable marker cassette and pNK003 backbone fragments were retrieved using the DNA clean kit with no need to separate them. The in-fusion online tool (https://www.takarabio.com/learning-centers/cloning/primer-design-and-other-tools, accessed on 24 June 2021) was used to design primers to amplify two homology arms with lengths of about 1.0 kb. The space between the two homology arms contained the target sequence and could not be longer than 50 bp to ensure that the DSB would be repaired with HR but not NHEJ [28]. Finally, the purified pNK003 backbone fragment, left homologous arm, hph marker cassette, and right homologous arm were mixed and assembled with the in-fusion method to obtain the pNK003/ADE2.E_HR plasmid containing the HR deletion structure, in which gDNA and Cas9 reside outside the homology arms and are resolved and degraded by double crossover during homologous recombination. This same approach is also applicable for the pRH003 plasmid used in the Serotype D strain. With this new method, the HR deletion structure plasmid could be constructed within one day.

### 2.5. Electroporation of Cryptococcus Species

Electroporation was performed using a previously reported protocol [29]. The yeast cells were incubated for about 3 h at 30 °C. Then, approximately 3–5 μg of purified linearized plasmids (less than 10 μL) from the desired plasmid constructs was added to the recipient cells in a 0.2 cm gap electroporation cuvette and electroporated at 1.4 kV. For the mutation of JEC21 *CFL2* and *CFL3* (two putative adhesion genes in *C. deneoformans*), the positive colonies were screened on YNB plates. For the mutation of *ADE2*, the positive colonies were screened on YNB plates with 20 mg L^−1^ adenine, as previously described [30]. For the transformants derived from *C. neoformans* H99, YPD plates containing 100 mg L^−1^ Hygromycin B were used.

### 2.6. Southern Blot Analysis

Overnight cultures grown to the stationary phase at 30 °C were harvested, washed, and subjected to genome DNA extraction, as described previously [18]. Appropriate restriction enzymes, as described in the figure legends, were utilized to digest approximately 10 μg of genomic DNA for each strain. The digested DNA was then subjected to separation on 0.8% agarose gel and transferred to N^+^-Magaprobe nylon transfer membranes following the manufacturer’s instructions (Dingguo, Beijing, China). The primers listed in Table S1 were used to amplify the probe sequences, and the DIG High Primer DNA Labeling and Detection Starter Kit II (Roche China, Shanghai, China) was applied to label the DNA probe by following the manufacturer’s instructions.

## 3. Results

### 3.1. Rapid Cloning of the Target Sequence into the All-In-One CRISPR-Cas9 Vector

**Figure 1:**
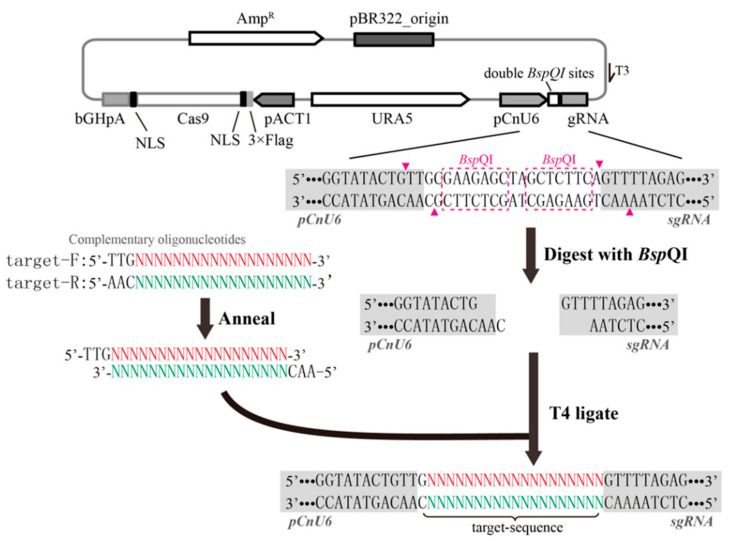
Schematic illustration of the method used to rapidly introduce a target sequence into the gRNA expression cassette in the new CRISPR-Cas9 vector. Three cassettes, including a selection marker, Cas9 nuclease, and a single gRNA with two reverse BspQI restriction sites, were included in the plasmid. After digestion using BspQI, a pair of annealed oligonucleotides containing 5′ compatible TTG and AAC overhangs were cloned scarlessly into the vector right before the sgRNA scaffold. The pink dotted lines show the sites for BspQI recognition, and BspQI digestion occurs at the sites of the pink triangles.

The rate-limiting step of the all-in-one CRISPR-Cas9 system in fungi is often the cloning pipeline of the target sequence into the proper locus in the gRNA expression cassette. In our previous studies, overlap PCR was adopted to introduce a target sequence, which is a complex and time-consuming cloning step [18]. In some papers investigating the use of the CRISPR-Cas9 system for *C. neoformans*, gRNA expression cassettes containing the target sequence were directly synthesized; however, this is expensive and not feasible for all researchers [23]. Here, we constructed a new gRNA expression cassette on the CRISPR-Cas9 vector with double BspQI restriction sites for simple target sequence cloning, instead of using overlap PCR. The most striking characteristic of the BspQI restriction endonuclease is that it cuts the DNA sequence outside the recognition site. Therefore, the ligation of two reverse BspQI sites between the CnU6 promoter and guide RNA enabled us to produce cuts with 5′ overhangs between them. The target sequence was introduced simply by annealing two hybridized oligos with complementary 5′ overhangs (Figure 1). Sequencing using a T3 universal primer was used to ascertain proper ligation of the target sequence. In this way, we successfully constructed two single gRNA expression cassettes for *C. neoformans* H99 and three other cassettes for *C. deneoformans* JEC21. The sequencing results revealed that more than 90% of clones randomly selected on selective media successfully introduced the target sequence into the gRNA expression cassette, indicating the high accuracy rate of gRNA construction using the new CRISPR-Cas9 vector.

### 3.2. High Efficiency of the Simplified All-In-One Vector in Gene Mutagenesis

We previously described the upfront all-in-one ‘suicide’ CRISPR-Cas9 vector in the *C. deneoformans* strain, but this has not yet been described in *C. neoformans*. Thus, to evaluate the targeting efficiency of the pNK003 system in the *C. neoformans* H99 strain, *ADE2* and *CAP64* were selected as the target genes due to the feasible phenotypes observed after gene mutation. For genome editing, we first downloaded the entire genome of the H99 strain from the GenBank database and, subsequently, applied sgRNAcas9 software to search for the optimal target sequence to minimize the probability of off-target effects. Following the development of this pipeline, the ADE2.Z target site for the *ADE2* gene and CAP64.Z for *CAP64* were selected and inserted into the gRNA expression cassette through hybridized oligos. Then, the linearized all-in-one CRISPR-Cas9 vector without donor DNA was transformed into wild-type H99 yeast cells via the electroporation method.

**Figure 2:**
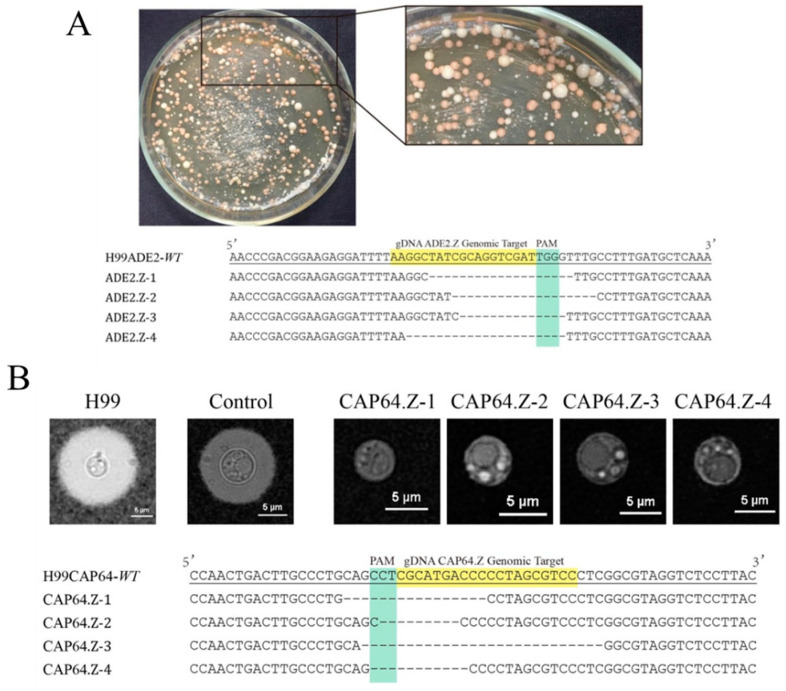
Target gene mutagenesis with the all-in-one CRISPR-Cas9 system expressed by the pNK003 vector. (**A**) *ADE2* mutagenesis was mediated by the pNK003/ADE2.Z plasmid in the H99 strain. The *ADE2*-deficient transformants exhibited pink clones on the YPD plate supplemented with 100 μg mL^−1^ hygromycin. Alignments of the *ADE2* gene from four randomly selected pink colonies revealed indels in the sequences. H99ADE2-WT is the wild-type reference *ADE2* gene sequence; ADE2.Z 1-4 are mutants from the population. The target and PAM sequence are highlighted. (**B**) *CAP64* mutagenesis was created by the pNK003/CAP64.Z plasmid in the H99 strain. Alignments of the *CAP64* gene from four randomly selected unoiled colonies revealed indels in the sequences. H99CAP64-WT is the wild-type reference *CAP64* gene sequence; CAP64.Z 1-4 are mutants from the population. The target and PAM sequence are highlighted. The morphological analysis of wild-type H99 cells and *cap64*∆ mutants confirmed the deficiency of *CAP64*.

*ADE2* encodes a phosphoribosylaminoimidazole carboxylase in the biosynthetic pathway of adenine, the loss-of-function of which often results in an adenine auxotroph that forms pink colonies on culture plates containing low levels of adenine [31]. Transformants that have been targeted and mutated by the CRISPR-Cas9 system were initially identified via their pink color on YNBA or YPD media. By introducing the linearized pNK003/ADE2.E plasmid into H99 through electroporation, a large proportion of Hygromycin B-resistant transformants formed pink colonies on the YNBA plates, as expected (Figure 2A), indicating that successful targeting had occurred at the ADE2.E locus by pNK003/ADE2.E. As a control, pink transformant was never obtained by introducing a linear empty pNK003 plasmid. Then, we randomly selected four pink colonies resulting from the transformation of pNK003/ADE2.E. The sequencing results showed that various indel mutations appeared at the ADE2.E locus in all four pink colonies (Figure 2A), preliminarily proving the efficiency of pNK003 in H99 gene mutagenesis.

**Figure 3:**
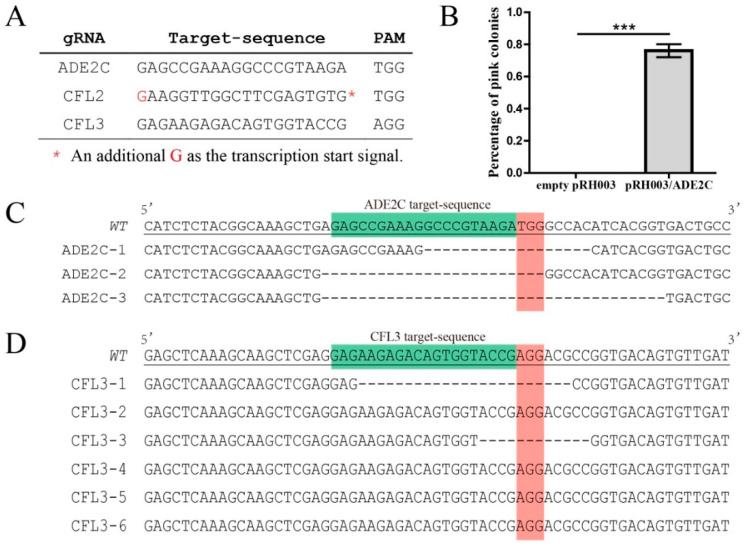
Target gene mutagenesis with the all-in-one CRISPR-Cas9 system expressed by the pRH003 vector. (**A**) Three target sequences and their PAM sequences are indicated. (**B**) The proportion of *ADE2* mutants generated with pRH003/ADE2C. The CRISPR-Cas9 system could not target ADE2C locus without the guidance of gRNA carrying the ADE2C target sequence. Only the *URA5* positive transformants containing gRNA, which carries the ADE2C target sequence, could form a large proportion of pink colonies. The boxes indicate the mean values, and error bars correspond to the SEM (three replicates per experiment). *** *p* < 0.001. (**C**) Alignments of the partial *ADE2* gene sequences from three pink colonies transformed with linear pRH003/ADE2C. WT is the wild-type reference *ADE2* gene sequence; ADE2C-1/-2/-3 are mutants from the population. Target and PAM sequences are highlighted. (**D**) Alignments of *CFL3* gene from six randomly selected colonies transformed with linear pRH003/CFL3. Indels were detected in two out of the six transformants. WT is the wild-type reference *CFL3* gene sequence; CFL3-1/-3 are mutants from the population. Target and PAM sequences are also highlighted.

To examine whether other genes in *C. neoformans* could be similarly disrupted by pNK003, we targeted another gene, *CAP64*, the disruption of which would apparently lead to a loss of the capsule as well as virulence [9]. Likewise, four transformants were randomly selected for phenotypic observation under a microscope and submitted for sequencing confirmation. The results indicated the formation of a frameshift mutation at the *CAP64* target locus in four transformants (Figure 2B). These results demonstrate that the pNK003 vector can cleave double-stranded DNA targets with high efficiency in the *C. neoformans* H99 strain and introduce indels by repairing DSBs with NHEJ. To further test the new CRISPR-Cas9 vector, pRH003, on the Serotype D strain, the same pipeline was formed using *ADE2* and *CFL3* as the candidate target genes. The results demonstrated the high efficiency of the simplified pRH003 vector in directing gene mutagenesis (Figure 3).

### 3.3. Knockout of Genes in One Step by the Simplified ‘Suicide’ CRISPR-Cas9 Vector

**Figure 4:**
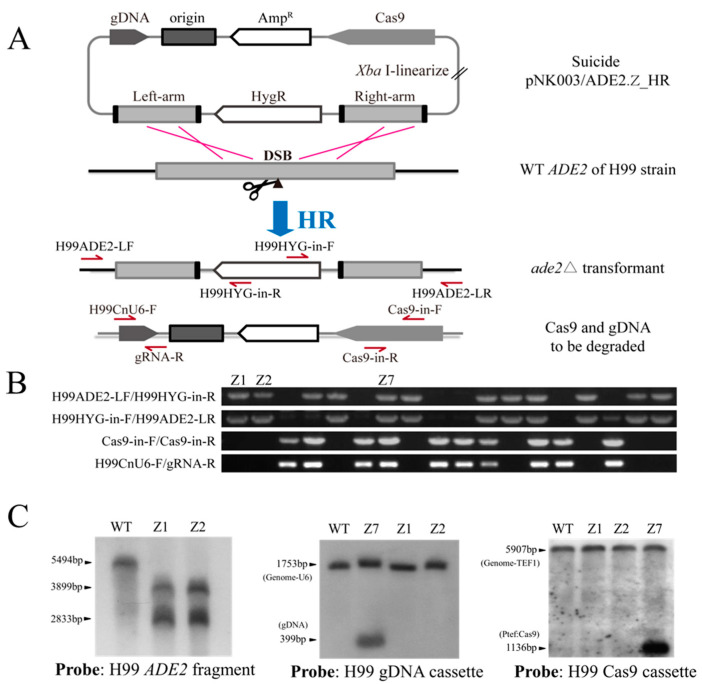
Disruption of the *ADE2* gene in the H99 strain with the pNK003 plasmid. (**A**) Construction scheme used to produce the suicide plasmid based on the pNK003 vector. The left and right arms can be assembled into pNK003/ADE2.Z simultaneously using in-fusion cloning technology. CRISPR-Cas9 cassettes outside HR fragments would be resolved after double crossover. The primers used for PCR detection are indicated with arrows. (**B**) Four groups of PCR amplification were performed to verify the HR disruption of *ADE2* (the two upper rows) and the elimination of Cas9 (the third row) and gDNA (the bottom row) in 18 pink transformants. The results show that 8/18 transformants had a disrupted *ADE2* gene, and both Cas9 and gDNA were eliminated. The specific primer pairs are indicated on the left side of the gel. (**C**) Southern blotting was conducted to confirm that *ADE2* was disrupted and CRISPR-Cas9 cassettes were eliminated. The left panel shows the disruption of *ADE2*, in which genomic DNA was digested with EcoRI. The control WT had a wild-type *ADE2* band (5494 bp), and the mutant strain had two bands (3899 bp and 2833 bp). The middle and the right panels show the loss of the gDNA and Cas9 cassettes in the Z1 and Z2 mutants.

Gene deletion and subsequent complementation are important tools that can be used to study the roles of genes in fungi to ensure that a direct relationship occurs between phenotypic consequences and artificial mutation [32]. After gene editing, the constitutive expression of the CRISPR-Cas9 system that integrates into the genome of a specific organism prevents the reintroduction of the wild-type gene, and the off-target effects likely accumulate with the increasing passage of cells. To eliminate Cas9 and gDNA cassettes, we have already constructed a ‘suicide’ CRISPR-Cas9 system with a cis arrangement of gDNA and Cas9 cassettes to one side of the homology arms of the deletion construct. However, during routine experiments, we noticed that selection of suitable restriction sites used to clone homology arms was often interfered with by restriction sites inside the homology arms. In addition, the two-step cloning procedure used to clone homology arms is time-consuming. Here, we adopted in-fusion cloning technology to ligate the pNK003/ADE2.Z plasmid fragment, 1.0 kb ADE2.E left arm, digested hph marker cassette, and 1.0 kb ADE2.E right arm to construct the pNK003/ADE2.Z_HR plasmid in one step (Figure 4A).

The linearized pNK003/ADE2.Z_HR vector was electroporated into the H99 strain. After reviving for 4 h, the transformed cells were spread on hygromycin-containing YPD plates for selection. Eighteen random selected colonies were used for PCR analysis, and the results show that double homologous crossover occurred in 11 out of 18 transformants, while degradation of Cas9 and gDNA cassettes occurred in 8 out of 11 transformants (Figure 4B and Figure S3). Furthermore, Southern blotting was applied to confirm the disruption of H99 *ADE2* and the degradation of CRISPR-Cas9 elements, including the Cas9 and gRNA expression cassettes (Figure 4C and Figure S4). Transformants Z1, Z2, and Z7 were selected according to the PCR results, and the genome was extracted. As expected, the Southern blotting results confirmed the disruption of *ADE2* and the loss of Cas9 and gDNA in the Z1 and Z2 transformants, whereas in the control transformant Z7, gDNA and Cas9 cassettes were indeed present (Figure 4C and Figure S4). Together, this demonstrates the availability of the pNK003_HR plasmid and its relation to the elevation of HR efficiency and the elimination of the CRISPR-Cas9 cassette after gene editing.

**Figure 5:**
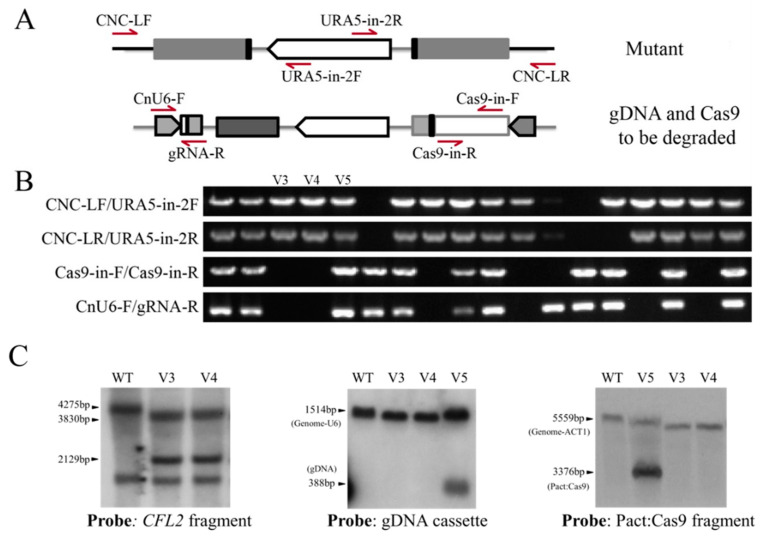
*CFL2* disruption mediated by the simplified pRH003 system. (**A**) The locations of all primers used for PCR verification are indicated by arrows. (**B**) Confirmation of *CFL2* disruption and CRISPR-Cas9 elimination by four rounds of PCR amplification in 18 transformants. The first and second rows confirm *CFL2* disruption, the third row confirms the loss of Cas9, and the bottom row confirms the loss of gDNA. The results show that 6/18 transformants had a disrupted *CFL2* gene, and both Cas9 and gDNA were eliminated. The specific primer pairs are indicated on the left side of the gel. (**C**) Southern blotting was conducted to confirm that *CFL2* was disrupted and CRISPR-Cas9 cassettes were eliminated. The left panel shows the disruption of *CFL2*, in which genomic DNA was digested with Hind III. The control 4500FOA had a wild-type *CFL2* band (4275 bp), and the mutant strain had two bands (3830 bp and 2129 bp). The middle and the right panels show the loss of the gDNA and Cas9 cassettes in the V3 and V4 mutants.

Likewise, to test whether genes in the *C. deneoformans* genome could be similarly disrupted as well as to test the efficiency of spontaneous elimination of the CRISPR-Cas9 system by using the pRH003 plasmid, we targeted the JEC21 *ADE2* and *CFL2* genes (Figure 5 and Figure S2). Based on the results identified previously, we first constructed the pRH003/ADE2C vector containing the ADE2C target locus, which resides in the coding region and has been proven to be targeted efficiently by the Cas9 protein [18]. In-fusion cloning technology was adopted to ligate the pRH003/ADE2C fragment, the 1.0 kb ADE2C left arm, the digested *URA5* cassette fragment, and the 1.0 kb ADE2C right arm to construct the pRH003/ADE2C_HR plasmid in one step. The PCR results confirmed the operability of new pRH003 vector in gene disruption via HR and the efficiency of CRISPR-Cas9 fragment elimination (Figure S2).

*CFL2*, a novel gene, was selected as the editing object for the first time and used to further confirm the homologous recombination triggered by the pRH003 vector with CRISPR-Cas9 elimination after editing. *CFL2* (CNC04160) is also a putative adhesion gene, and disruption of it did not result in significant phenotype variation on YNB plates. After transformation, 18 colonies were randomly selected for PCR analysis, and the results showed that double crossover occurred in 14 out of 18 transformants. In six out of fourteen transformants, Cas9 and gDNA cassettes were degraded (Figure 5B and Figure S5). The Southern blot results confirmed the disruption of *CFL2* and the loss of Cas9 and gDNA in V3 and V4 transformants, whereas in the control transformant V5, gDNA and Cas9 cassettes were indeed present (Figure 5C and Figure S6). Similarly, 18 control transformants obtained by electroporation with only the CFL2_HR deletion structure but not CRISPR-Cas9 factors were randomly selected to verify the occurrence of homologous recombination by two groups of PCR tests. However, none of the predicted bands were observed in the eighteen picked transformants. These experimental results demonstrate that the pRH003_HR vector could dramatically increase the efficiency of HR by introducing DSBs into the genome of *C. deneoformans*. Cas9 and gDNA cassettes located outside the HR construct could be degraded during double crossover, similar to the effect of pBS-URA5-CRISPR_HR [18].

## 4. Discussion

In recent years, the breakthrough development of the CRISPR-Cas9 genome editing system has shown great potential to accelerate the field of fungal research by widely contributing to the interpretation of gene functionality. In 2013, Dicarlo et al. used the CRISPR-Cas9 genome editing system in *S. cerevisiae* as the first application of this technology for genetic manipulation in fungi [21]. Subsequently, this gene editing technology has been widely applied in several strains, including the model filamentous fungi *Neurospora crassa* and *Aspergillus nidulans* [33,34], the free-living yeasts *S. pombe* [22] and *Candida glabrata* [35], as well as the opportunistic fungal pathogen *Cryptococcus* species. For fungi that are accessible through electroporation transformation or biolistic methods, different plasmids separately expressing Cas9, gRNA, or donor DNA can be utilized to introduce the editing system into cells. For example, as the first application in fungi, DiCarlo et al. transformed a haploid yeast with two plasmids separately expressing SpCas9 and gRNA [21]. For fungi that are difficult to transform with these methods, Agrobacterium-mediated transformation (AMT) may be applied. In this method, T-DNA integrates predominantly as a single copy, and thus, this mainly excludes the possibility of co-transformation [36]. Considering the technological disparity among diverse fungi, the all-in-one CRISPR-Cas9 system, which contains all the functional elements needed for genome editing in one plasmid, was constructed in our lab.

To improve the homologous recombination frequency and facilitate genetic studies of *Cryptococcus* species, we established the ‘suicide’ CRISPR-Cas9 gene editing system in *C. deneoformans*. It was designed in the all-in-one form to contribute to its widespread application among different fungi, but it has not yet been used in the *C. neoformans* strain. In the *C. deneoformans* JEC21 strain, human-codon-optimized Cas9 is driven by a promoter of the cryptococcal actin-encoding gene ACT1 and ends with a bGHpA terminator. On the same plasmid, the RNA Polymerase III promoter of the U6 small RNA gene is used for the transcription of gRNA [18]. The ‘suicide’ CRISPR-Cas9 construction is highly efficient when used in the JEC21 strain. However, the time-consuming and sophisticated procedures required to construct vectors impede its widespread application. Here, we reported the optimized and simplified construction of ‘suicide’ CRISPR-Cas9 vectors in both *C. neoformans* and *C. deneoformans* strains, correspondingly named pNK003 and pRH003.

The design of two reverse BspQI restriction sites between the CnU6 promoter and gRNA structural component made the process of introducing target sequences simple, rapid, and extremely efficient. In fact, when a BspQI-cut linear plasmid that could be stored at −20 °C for long periods of time was prepared, the only procedure required to clone target sequences was the introduction of hybridized oligos into the gRNA expression cassette by the T4 DNA ligase reaction. Only two pairs of primers and one round of PCR are needed to generate CRISPR-Cas9. In order to ensure the degradation of the CRISPR-Cas9 system after gene editing, the HR deletion structure needed to be outside Cas9 and gDNA. To this end, a new cloning method using in-fusion cloning technology was set up to ligate two homology arms around the target site at the same time and avoid interference by restriction sites in the homology arms. This method is much more convenient and faster during the introduction of two homology arms than the traditional method.

Our results indicate that the efficiency of producing indels and the HR structure and subsequent spontaneous elimination of the CRISPR-Cas9 system with the pRH003 or pNK003 systems are very similar to what has been reported using pBS-URA5-CRISPR. However, hybridized oligo-based construction of the gRNA expression cassette is much simpler. The introduction of in-fusion cloning technology greatly shortened the time needed to construct the HR deletion structure and excluded constraints by restriction sites. Except for the gene manipulations mentioned above, these CRISPR-Cas9 vectors have been widely used in our lab. Via the rough statistics, more than 60 genes have been disrupted by different operatives with these all-in-one CRISPR-Cas9 vectors. Hopefully, the availability of these new simplified CRISPR-Cas9 plasmids could further broaden the use of this system and help to accelerate functional genomics studies of *Cryptococcus* species.

## Data Availability

Not applicable.

## Supplementary Materials

The following are available online at https://www.mdpi.com/article/10.3390/jof7070505/s1, Table S1: primers used in this study, Figure S1: map of the plasmids, Figure S2: disruption of the ADE2 gene with the newly constructed pRH003 system, Figure S3: full-length gel results of Figure 4B, Figure S4: full-length blot results of Figure 4C, Figure S5: full-length gel results of Figure 5B, Figure S6: full-length blot results of Figure 5C, Figure S7: full-length gel results of Figure S2D.
