# Supplementary material for: Simplified All-In-One CRISPR-Cas9 Construction for Efficient Genome Editing in Cryptococcus Species
Source: J Fungi (Basel). 2021 Jun 24;7(7):505. doi: 10.3390/jof7070505 (PMC8303259; doi:10.3390/jof7070505)
Supplement: Supplementary file 1 [file jof-07-00505-s001.zip › 2. supplementary materials.pdf]

Table S1. Primers used in this study

Primers	Sequences
pX-F	CCCTATCTCGGGCTATTCTT
pX-R	AAAAGGCCAGGAACCGTAAA
pT3-F	GGTTCCTGGCCTTTTAATTAACCCTCACTAAAGGG
pT7-R	TAGCCCGAGATAGGGGTAATACGACTCACTATAGG
CnU6-F	CCATCGATTTGCATTAGAACTAAAAACAAAGCA
BspQI-ZU6-R	AACTGAAGAGCTAGCTCTTCGCAACAGTATACCCTGCCGG TG
BspQI-gRNA-F	TTGCGAAGAGCTAGCTCTTCAGTTTTAGAGCTAGAAATAG CAAGTT
gRNA-R	CCGCTCGAGTAAAACAAAAAAGCACCGAC
pTEF1-F	ATCCACTAGTTCTAGACGGTACAAGATTGTGGCTACTA
pTEF1-R	TCCTTATAGTCCATGGTTTGAAGTTTTCTGTGGAGATCGT
H99CnU6-F	ATATCAAGCTTATCGATTCCGAACATTGCATAAATTTGCA
BspQI-H99CnU6-R	AACTGAAGAGCTAGCTCTTCGCAACAGTATATACACCTCG TGAA
H99HYG-cassette-F	TCGGATCGATAAGCTTCTGCGAGGATGTGAGCTGGA
H99HYG-cassette-R	TAGAACTAGTGGATCCGAAGAGATGTAGAAACTAGCTTC
H99ADE2.Z-F	TTGAAGGCTATCGCAGGTCGAT
H99ADE2.Z-R	AACATCGACCTGCGATAGCCTT
H99CAP64.Z-F	TTGGACGCTAGGGGGTCATGCG
H99CAP64.Z-R	AACCGCATGACCCCCTAGCGTC
H99ADE2-LF	TCCAGATAGGGGACCAGATG
H99HYG-in-R	AAAGCACGAGATTCTTCGCC
H99HYG-in-F	GTCCGAGGGCAAAGGAATAG
H99ADE2-LR	TGTTAAGGGCGGGTACAGAT
H99ADE2-probe-F	TGAGCAGCTTACCCTTGGTA
H99ADE2-probe-R	TGAGGTCGATCCAGTCTGCA
H99gDNA-probe-F	TCCGAACATTGCATAAATTTGC
gDNA-probe-R	TAAAACAAAAAAGCACCGAC
H99cas9-probe-F	ATGGTCGCAAGTGTGGTT
Pact:Cas9 Probe-R	TCAGGTGGTAGATGGTGGG
ADE2C-target-F	TTGAGCCGAAAGGCCCGTAAGA
ADE2C-target-R	AACTCTTACGGGCCTTTCGGCT
ADE2-cexu-F	CCCAGTCCCCAAACTATCA
ADE2-cexu-R	GCATAACGGGCAAGTCAG
ADE2C-up-F	TTCTAATGCAAATCGATCCTCGACTCTGGCTCCT

ADE2C-up-R	ATATCAAGCTTATCGATCTCAGCTTTGCCGTAGAG
ADE2C-down-F	AGCGAACGTGGGATCCACATCACGGTGACTGCCGA
ADE2C-down-R	TAGAACTAGTGGATCACCCGACATCAAACCAACAC
ADE2C-LF	CAGCAGCTTCAGTCATGCA
ADE2C-LR	TGTCGATGGCAATCGTGTC
CFL3-target-F	TTGAGAAGAGACAGTGGTACCG
CFL3-target-R	AACCGGTACCACTGTCTCTTCT
CFL3-cexu-F	AGTTTCGTCACCGCAAGAGC
CFL3-cexu-R	CGTCCACTATTGCGACTTTG
CFL2-target-F	TTGAAGGTTGGCTTCGAGTGTG
CFL2-target-R	AACCACACTCGAAGCCAACCTT
CFL2-up-F	TTCTAATGCAAATCGCACCGGACGCTAAGGAAACC
CFL2-up-R	ATATCAAGCTTATCGGCCAACCTTGAACCAGTCGG
CFL2-down-F	AGCGAACGTGGGATCTGAAACAGAATTGAACTCTTGC
CFL2-down-R	TAGAACTAGTGGATCCTATGACGAGCGGCGTTTTG
CFL2-LF	CGGGTTTAGGAATGTGAAGG
CFL2-LR	TGACCATAGCGACCGAGAAT
T3	AATTAACCCTCACTAAAGGG
Cas9-in-F	CCCACCATCTACCACCTGA
Cas9-in-R	GCCGCCAGTAGTTCTTCAT
URA5-in-2F	CGATGACTCCCTGGTCCCAT
URA5-in-2R	CACCGTGGATCATGTCGATC
CFL2-probe-F	CACCGGACGCTAAGGAAACC
CFL2-probe-R	CTATGACGAGCGGCGTTTTG
FOAgDNA-probe-F	TTGCATTAGAACTAAAAACAAAGCA
gDNA-probe-R	TAAAACAAAAAAGCACCGAC
Pact:Cas9 Probe-F	GGTGAGTCATCTTCCCGTCC

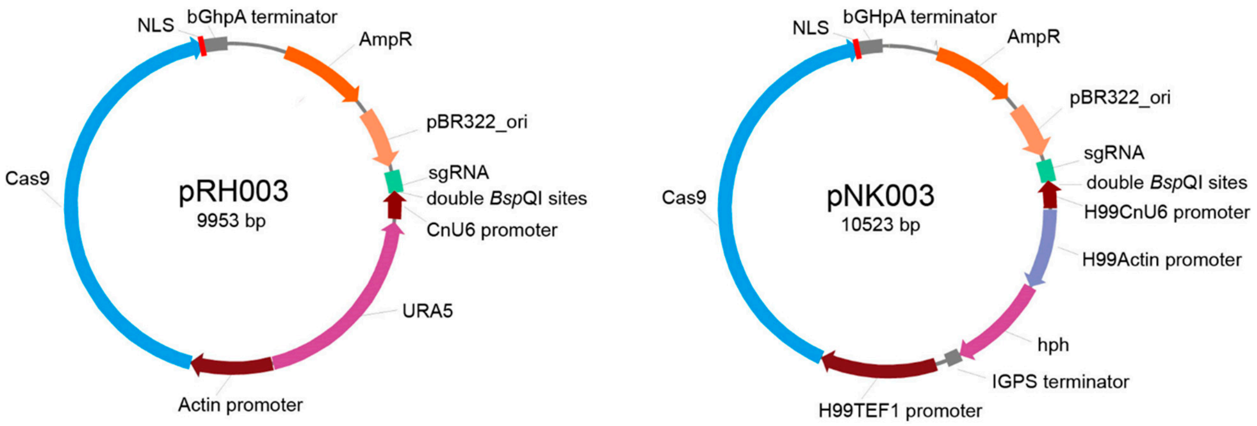

Figure S1 (**A**) Map of plasmid pRH003. (**B**) Map of plasmid pNK003.

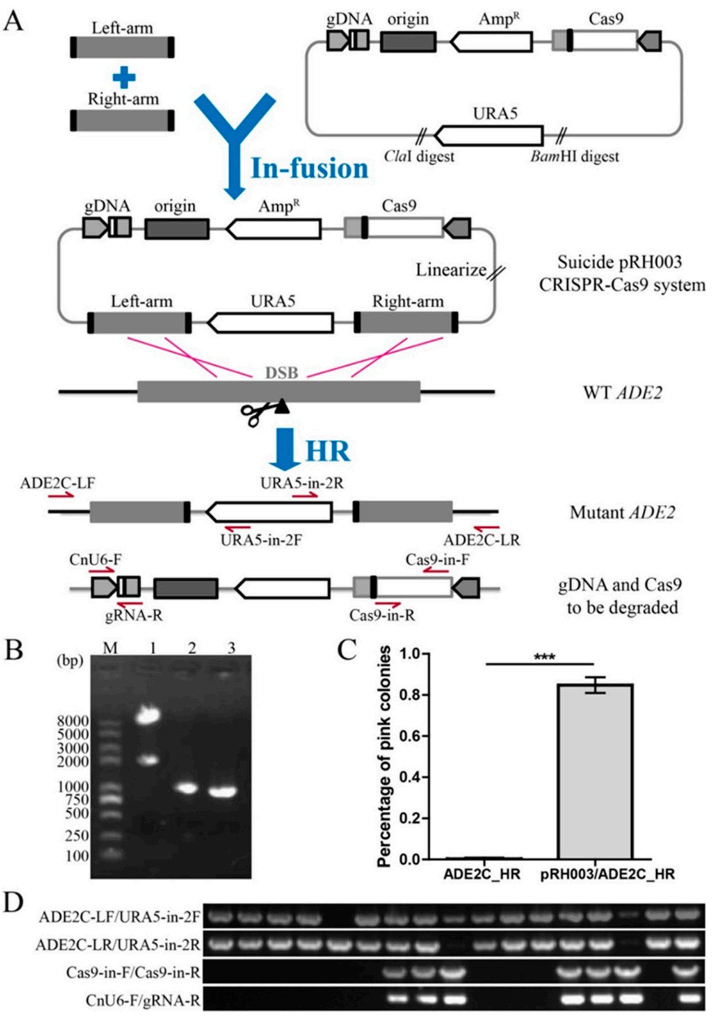

Figure S2. Disruption of *ADE2* gene with the newly constructed pRH003 system. (**A**) Construction scheme of the suicide plasmid based on pRH003. CRISPR-Cas9 cassettes outside HR fragments would be resolved after double crossover. The primers used for PCR detection are indicated with arrows. (**B**) Electrophoretogram of four

segments used for assembling pRH003/ADE2C_HR plasmid. M. Trans2K plus II marker; 1. pRH003 backbone (long) and *URA5* cassette (short); 2. Left *ADE2C* homologous arm; 3. Right *ADE2C* homologous arm. (**C**) Percentage of pink colonies on the YNBA plates. ADE2C_HR is the HR structure of the *URA5* marker flanked by 1.0 kb ADE2C homologous arms without CRISPR-Cas9 system. pRH003/ADE2C_HR represents the linearized plasmid which has a suicide CRISPR-Cas9 system targeting ADE2C. Boxes indicate mean values and error bars correspond to the SEM (three replicates per experiment). $^{***}P<0.001$. (**D**) Four groups of PCR amplification were performed to verify the HR disruption of *ADE2* (the two upper rows) and the elimination of Cas9 (the third row) and gDNA (the bottom row) in 17 pink transformants. The results show that 9/17 transformants had a disrupted *ADE2* gene, and both Cas9 and gDNA had been eliminated. The specific primers pairs are indicated on the left side of the gel.
