# Supplementary material for: Simplified All-In-One CRISPR-Cas9 Construction for Efficient Genome Editing in Cryptococcus Species
Source: J Fungi (Basel). 2021 Jun 24;7(7):505. doi: 10.3390/jof7070505 (PMC8303259; doi:10.3390/jof7070505)

Figure S3
Full-length gel results of Figure 4B.

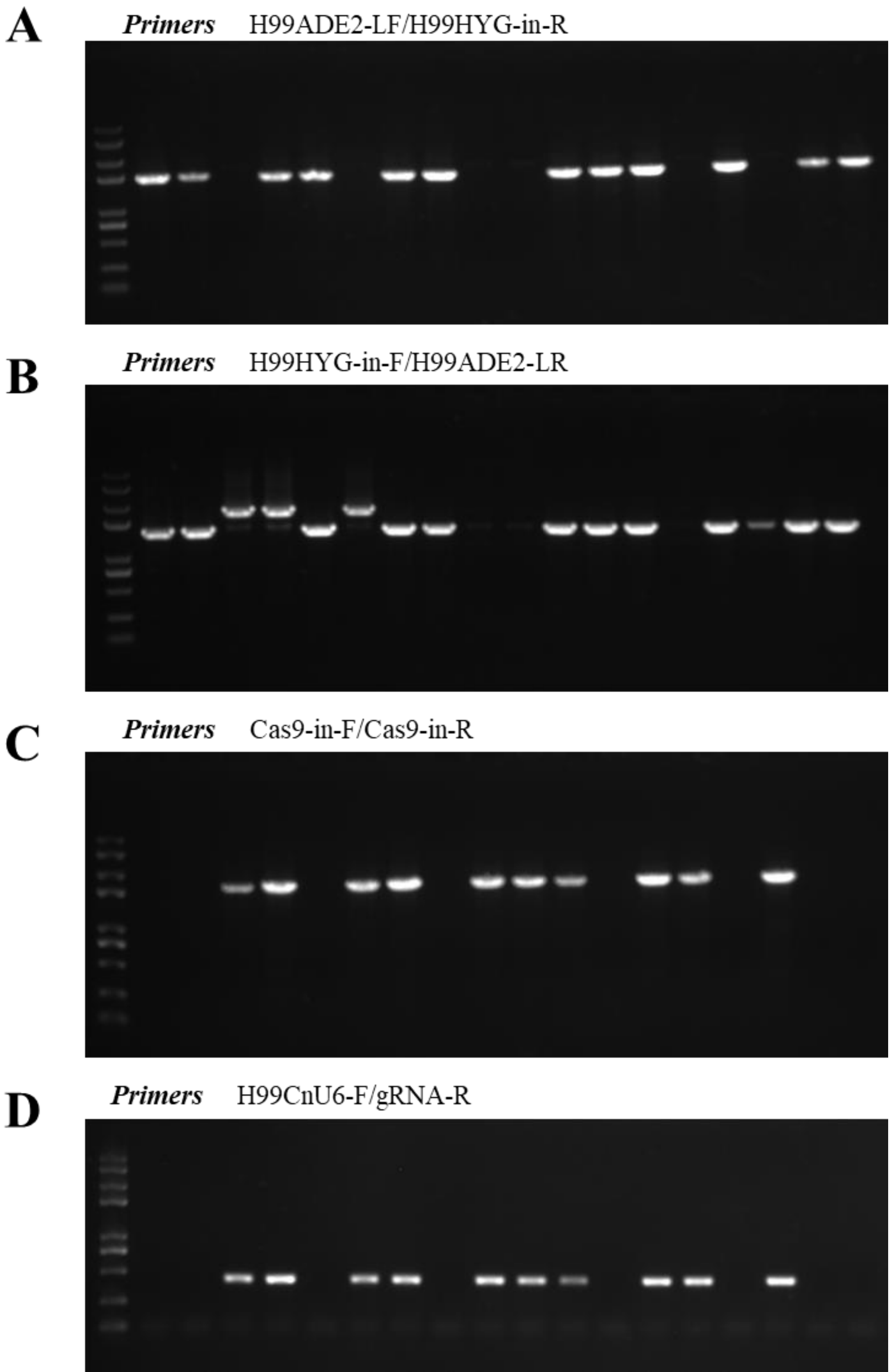

Figure S4 Full-length blot results of Figure 4C.

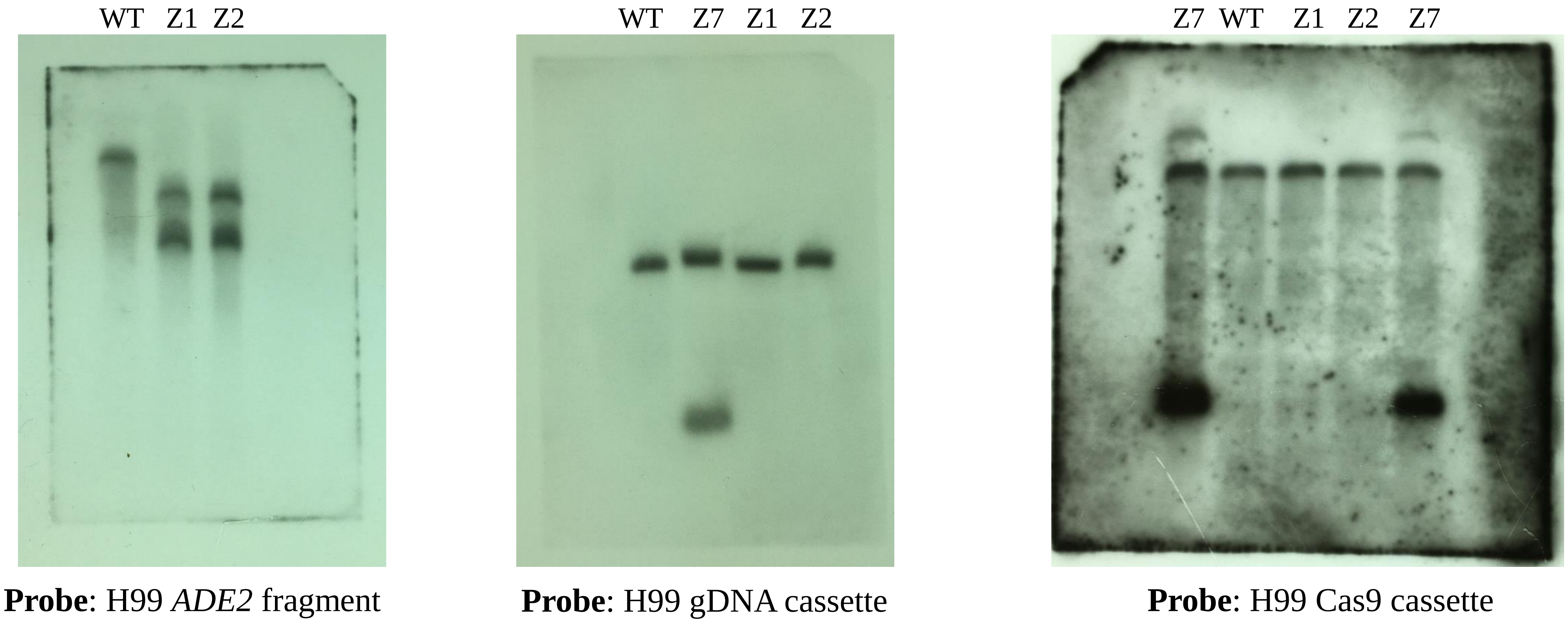

Figure S5
Full-length gel results of Figure 5B.

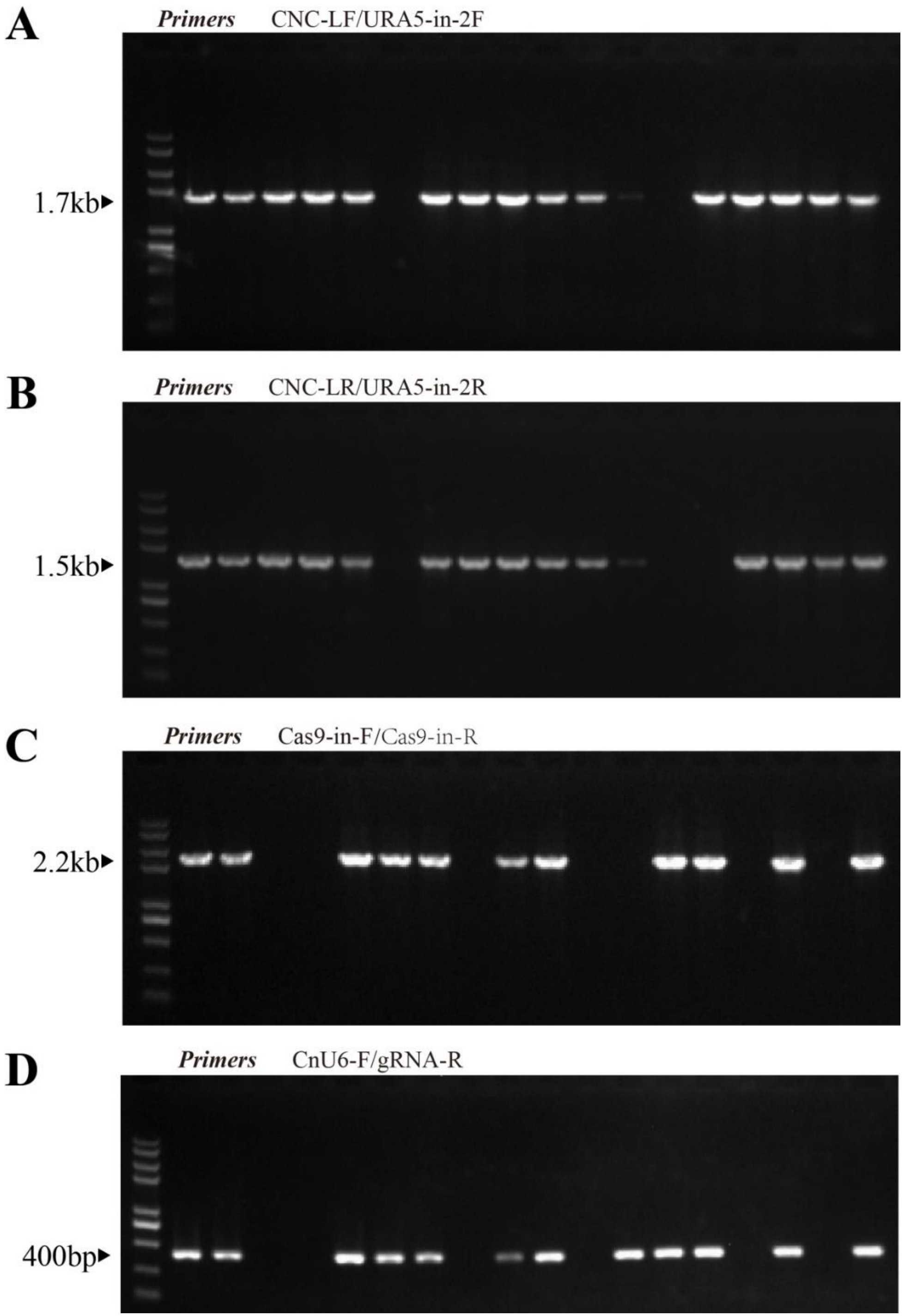

Figure S6

Full-length blot results of Figure 5C.

WT V3 V4 V5

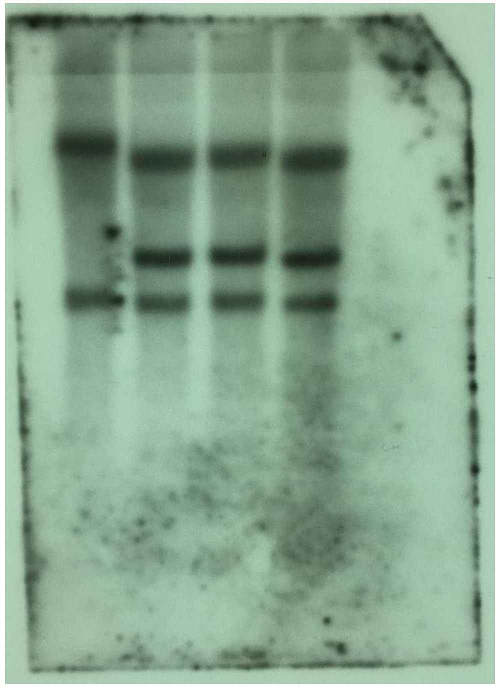

Probe: *ADE2* fragment

WT V3 V4 V5

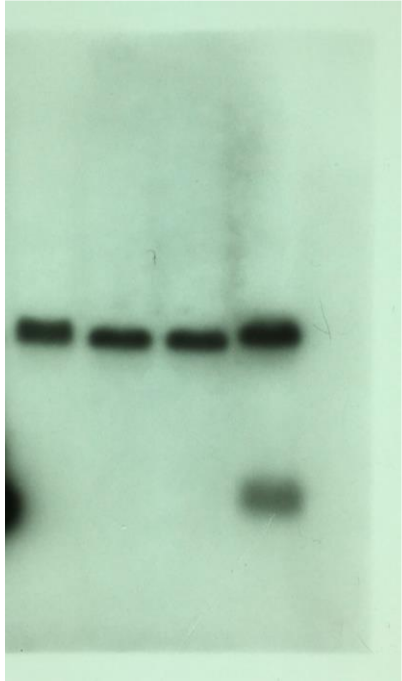

Probe: gDNA cassette

WT V5 V3 V4

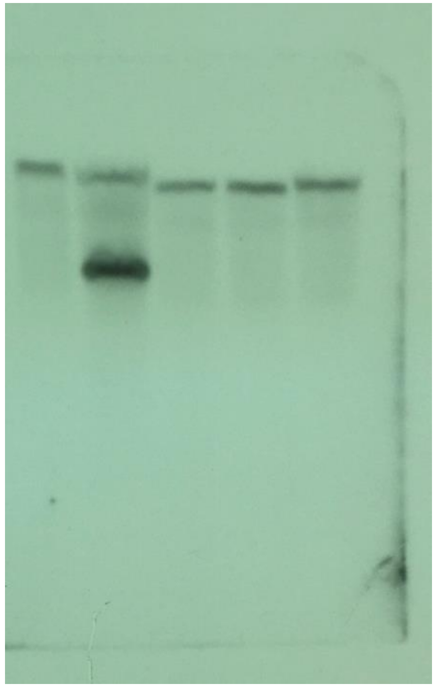

Probe: Pact:Cas9 fragment

Figure S7
Full-length gel results of Figure S2D.

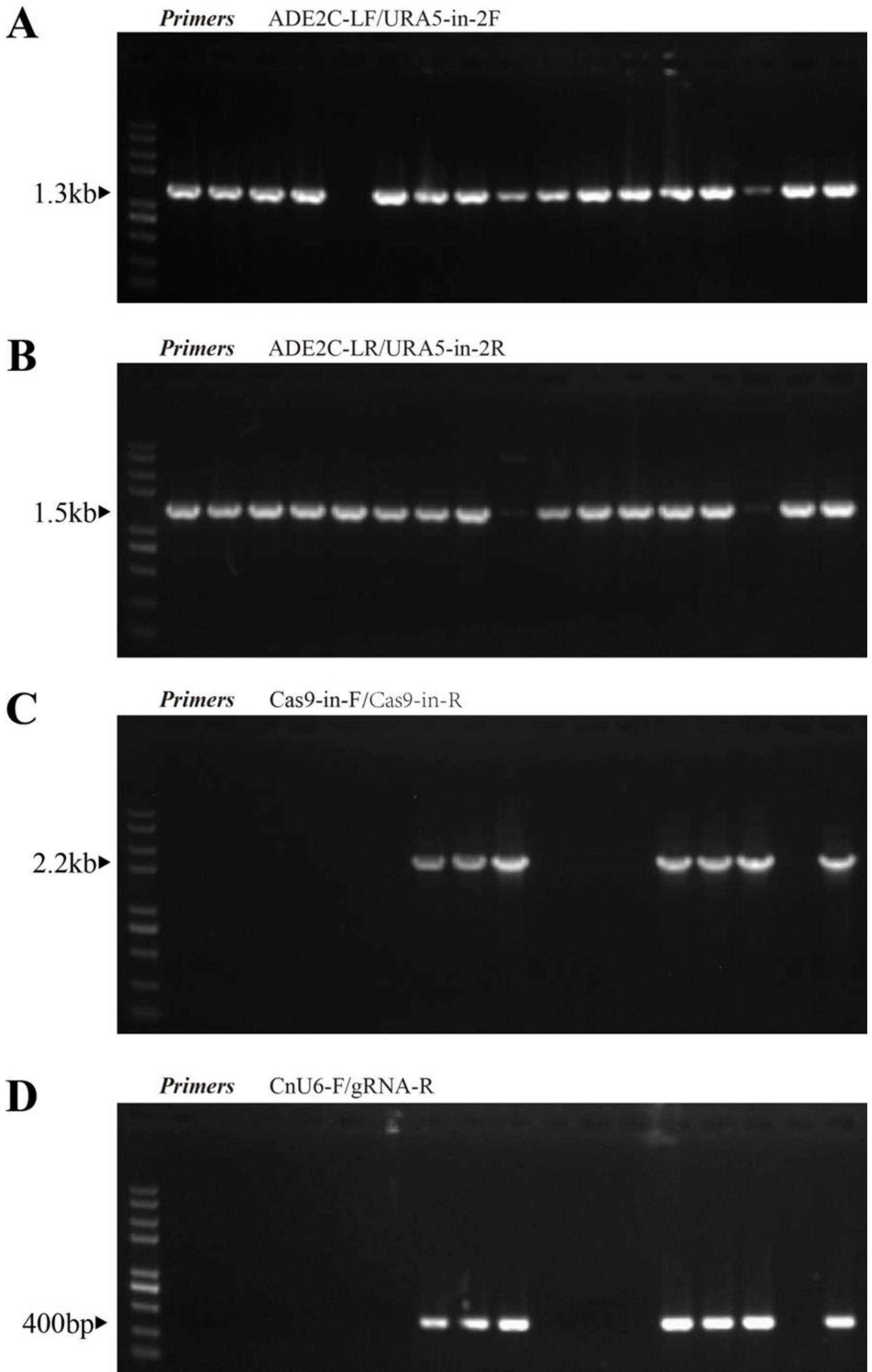

Supplement: Supplementary file 1 [file jof-07-00505-s001.zip › jof-1220012 suppl. figures.pdf]
